# Association of Education With Dementia Incidence Stratified by Ethnicity and Nativity in a Cohort of Older Asian American Individuals

**DOI:** 10.1001/jamanetworkopen.2023.1661

**Published:** 2023-03-06

**Authors:** Eleanor Hayes-Larson, Ryo Ikesu, Joseph Fong, Taylor M. Mobley, Gilbert C. Gee, Ron Brookmeyer, Rachel A. Whitmer, Paola Gilsanz, Elizabeth Rose Mayeda

**Affiliations:** 1 Department of Epidemiology, Fielding School of Public Health, University of California, Los Angeles; 2 Department of Community Health Sciences, Fielding School of Public Health, University of California, Los Angeles; 3 Department of Biostatistics, Fielding School of Public Health, University of California, Los Angeles; 4 Department of Public Health Sciences, University of California, Davis School of Medicine, Sacramento; 5 Alzheimer’s Disease Center, University of California, Davis Health, Sacramento; 6 Division of Research, Kaiser Permanente Northern California, Oakland

## Abstract

**Importance:**

High education protects against dementia, but returns on educational attainment may be different across sociodemographic groups owing to various social factors. Asian American individuals are a growing and diverse group, but little research has assessed dementia determinants in this population.

**Objective:**

To examine the association of education with dementia in a large cohort of Asian American individuals, stratifying by ethnicity and nativity.

**Design, Setting, and Participants:**

This cohort study used electronic health record (EHR) and survey data from the Research Program on Genes, Environment, and Health and the California Men’s Health Study surveys (2002-2020). Data are from Kaiser Permanente Northern California, an integrated health care delivery system. This study used a volunteer sample who completed the surveys. Participants included Chinese, Filipino, and Japanese individuals who were aged 60 to less than 90 years without a dementia diagnosis in the EHR at the time of the survey (baseline) and who had 2 years of health plan coverage before baseline. Data analysis was performed from December 2021 to December 2022.

**Exposures:**

The main exposure was educational attainment (college degree or higher vs less than a college degree), and the main stratification variables were Asian ethnicity and nativity (born in the US or born outside the US).

**Main Outcomes and Measures:**

The primary outcome was incident dementia diagnosis in the EHR. Dementia incidence rates were estimated by ethnicity and nativity, and Cox proportional hazards and Aalen additive hazards models were fitted for the association of college degree or higher vs less than a college degree with time to dementia, adjusting for age (timescale), sex, nativity, and an interaction between nativity and college degree.

**Results:**

Among 14 749 individuals, the mean (SD) age at baseline was 70.6 (7.3) years, 8174 (55.4%) were female, and 6931 (47.0%) had attained a college degree. Overall, among individuals born in the US, those with a college degree had 12% lower dementia incidence (HR, 0.88; 95% CI, 0.75-1.03) compared with those without at least a college degree, although the confidence interval included the null. The HR for individuals born outside the US was 0.82 (95% CI, 0.72-0.92; *P* = .46 for the college degree by nativity interaction). The findings were similar across ethnicity and nativity groups except for Japanese individuals born outside the US.

**Conclusions and Relevance:**

These findings suggest that college degree attainment was associated with lower dementia incidence, with similar associations across nativity. More work is needed to understand determinants of dementia in Asian American individuals and to elucidate mechanisms linking educational attainment and dementia.

## Key Points

**Question:**

Does the association of education with dementia differ by ethnicity and nativity among Asian American individuals?

**Findings:**

In this cohort study of 14 749 Asian American individuals who were members of a large integrated health care delivery system, college degree attainment was associated with lower dementia incidence, with no difference by nativity. Associations varied across Asian ethnicities but were less precise.

**Meaning:**

These findings suggest that college degree attainment was associated with lower dementia incidence, with similar associations across nativity, but more work is needed to understand determinants of dementia in Asian American individuals.

## Teaser

This cohort study examines the association of education with dementia in a large cohort of Asian American individuals, stratifying by ethnicity and nativity.

## Introduction

Low education is a robust and modifiable factor associated with the risk of dementia.^1,2,3,4^ However, the health benefits of higher educational attainment may be different across sociodemographic groups.^5^ These differential returns may be due to processes such as resource substitution (in which the disadvantaged group benefits more from education because other health-preserving resources are less available to them) or opportunity constraints (in which the disadvantaged group benefits less from education because the opportunities afforded by it are more restricted because of such factors as structural and interpersonal racism).^5,6^ In dementia, returns on education are further impacted by the contribution of education to cognitive reserve and resilience, which may vary according to individual educational experiences.^6,7^ Understanding variability in the impact of educational attainment on dementia incidence is important to understand and mitigate sources of racial and ethnic disparities in dementia incidence and inform prevention strategies for all groups.^6,8^

Asian American individuals are a diverse and growing group who are critically understudied in health research. Some evidence suggests that the incidence of dementia among Asian American individuals is lower than that among most other racial and ethnic groups,^9,10^ but there is little evidence on determinants of dementia, including the impact of education, among Asian American individuals as a whole or in specific ethnic groups. Furthermore, Asian American individuals born outside the US may experience distinct educational and social trajectories that affect the potential health-related benefits of education.^11^ For example, bilingualism could yield additional protection against dementia, whereas more limited labor market opportunities for immigrants vs individuals born in the US with the same educational attainment could reduce benefits.^12,13,14,15,16,17^ A large proportion of Asian American individuals are born outside the US, with substantial heterogeneity across ethnic groups.

The aim of this cohort study was to evaluate the association of education with dementia incidence by Asian ethnicity and nativity, to evaluate potential differential associations of education and dementia incidence across these groups. We hypothesized that education would be protective against dementia for all groups, but more weakly for Asian American individuals born outside the US, consistent with opportunity constraints.

## Methods

### Study Design and Sample

This study analyzed a cohort of 184 929 White and Asian members of Kaiser Permanente Northern California (KPNC), an integrated health care delivery system. Participants were aged 60 to less than 90 years and had 2 years of prior continuous health plan coverage at the time they completed 1 of 2 health surveys: either the California Men’s Health Study (administered 2002-2003)^18^ or the Kaiser Permanente Research Program on Genes, Environment, and Health Survey (administered 2007-2009).^19,20^ The analytic sample was restricted to 15 004 individuals reporting Chinese, Filipino, and Japanese ethnicities (see next section for how race and ethnicity were defined) because these Asian ethnic groups had sufficient sample size of both individuals born in the US and those born outside the US. Other Asian ethnicities all had fewer than 15 dementia cases in at least 1 nativity stratum, resulting in unstable estimates, and were not included in the analyses. Participants were excluded if they had a prior electronic health record diagnosis of dementia (Alzheimer disease, vascular, and nonspecific dementias) at the time of their survey.

Written informed consent for survey participation was documented by KPNC at return of the completed survey. Analysis of the deidentified data was approved by the University of California, Los Angeles institutional review board. This manuscript follows the Strengthening the Reporting of Observational Studies in Epidemiology (STROBE) reporting guideline^21^ and the RECORD reporting guidelines^22^ for the use of routinely collected health data.

### Race and Ethnicity

Race and ethnicity were self-reported in the survey questions asking participants to mark all groups that best described their race or ethnicity. Asian American individuals were classified by the Asian ethnicities they reported (Asian Indian/South Asian, Chinese, Filipino, Japanese, Korean, and Vietnamese or other Southeast Asian). Participants who reported 1 Asian ethnicity with or without another non-Asian race or ethnicity were classified as the Asian ethnicity they reported, and participants who endorsed multiple Asian ethnicities (with or without a non-Asian race or ethnicity) were classified as multiethnic Asian.

### Primary Exposure: Educational Attainment

The main exposure was educational attainment, self-reported from the surveys. Participants were asked, “What is the highest level of school that you have completed?” with response options “Grade school (grades 1-8),” “Some high school (grades 9-11),” “High school or GED [general educational development],” “Technical/trade school,” “Some college,” “College,” or “Graduate school.” Because of the small sample sizes in some strata, we analyzed educational attainment dichotomously as college degree or higher vs less than college degree. A sensitivity analysis also examined educational attainment trichotomized as less than high school degree; high school degree, GED, or some college; and college degree or higher.

### Primary Outcome: Age at Dementia Onset

We defined incident dementia diagnoses during follow-up using *International Classification of Diseases, Ninth Revision (ICD-9)* and *International Statistical Classification of Diseases and Related Health Problems, Tenth Revision* codes (eTable 1 in Supplement 1) for Alzheimer disease, vascular dementia, and nonspecific dementia, and extracted diagnoses from electronic health records for inpatient, emergency, and outpatient settings, excluding laboratory-only and radiology-only encounters. A similar battery of *ICD-9* codes was reported to have a sensitivity of 77% and a specificity of 95% compared with a consensus diagnosis of dementia in a health care system in Seattle, Washington.^23^ In Medicare claims data, the use of a similar battery of *ICD-9 *codes for identifying cases had a sensitivity of 87% in a sample of patients with Alzheimer disease who participated in the Consortium to Establish a Registry for Alzheimer’s Disease.^24^ Follow-up was censored for participant death, lapse in health plan coverage 90 days or longer, or end of study period. Deaths were identified from the KPNC mortality database, which aggregates data from KPNC clinical and administrative sources, the National Death Index, California State death records, and Social Security Administration records. Because all event ages after 90 years were top-coded to deidentify the data, we used imputed event times after age 90 years, as previously reported for this cohort.^25^

### Additional Measures

We were interested in effect measure modification by nativity, which was self-reported in the KPNC surveys with the question, “Were you born in the United States?” (yes or no). Potential confounders obtained from electronic health records included age at survey completion, sex, and height (see eAppendix 1 in Supplement 1 for information for details on height data cleaning). We considered height a proxy for a number of early-life constructs, including nutritional environment and childhood socioeconomic status, and it is associated with cognitive outcomes.^26,27,28^ To describe the sample, we also report household size, household size–adjusted income, marital status, self-reported health, and smoking status from the KPNC surveys, but do not adjust for these variables, because they may be mediators of the effect of educational attainment on dementia incidence.

### Statistical Analysis

 Data analysis was performed from December 2021 to December 2022. All analyses were conducted for the sample overall and stratified by Asian ethnicity. We first estimated dementia incidence rates (crude and age-standardized using the 2000 US Census population aged ≥60 years as the standard) by nativity and educational attainment. We used Cox proportional hazards to estimate hazard ratios (HRs) and Aalen additive hazards models to estimate hazard differences (HDs) for the association between education and dementia incidence. Models were adjusted for age using age as the timescale (starting at survey age) and also were adjusted for sex. To assess for effect measure modification by nativity, all models included an interaction term between education and nativity; statistical tests for interaction were 2-sided, and *P* < .05 was used as the a priori level of significance. Sensitivity analyses included (1) models additionally adjusted for height, (2) models using time on study as time scale and controlling for baseline survey age, and (3) models using a 3-level version of education as described already.

Missing data were handled with multiple imputation (eAppendix 2 and eTable 2 in Supplement 1).^29,30^ Analyses were conducted using R statistical software version 4.1.3 (R Project for Statistical Computing). All statistical code is available elsewhere.^31^

## Results

The sample of 14 749 individuals included 6415 Chinese, 5020 Filipino, and 3314 Japanese individuals. The mean (SD) age at baseline was 70.6 (7.3) years, and the mean (SD) follow-up time was 9.9 (4.6) years. Overall, 55.4% of the sample (8174 individuals) was female, and 47.0% (6931 individuals) had attained a college degree. Filipino individuals were more likely to have a college degree (2758 individuals [54.9%] vs 2818 Chinese individuals [43.9%] and 1355 Japanese individuals [40.9%]) (Table 1) and to be born outside the US (4381 Filipino individuals [87.3%] vs 4312 Chinese individuals [67.2%] and 929 Japanese individuals [28.0%]). Other sociodemographic variables at baseline also varied by ethnicity. For example, Filipino individuals were less likely to be at least 65 years old (ie, Medicare eligible), household income per person was highest among Japanese individuals, household size was largest among Filipino individuals, and Chinese individuals were most likely to be married or living as married. Sample characteristics after multiple imputation and stratified by educational attainment and nativity are shown in eTables 3 and 4 in Supplement 1.

**Table 1.** Baseline Characteristics of the Sample Prior to Imputation, Stratified by Ethnicity

Characteristic	Participants, No. (%)			
	Overall (N = 14 749)	Chinese (n = 6415)	Filipino (n = 5020)	Japanese (n = 3314)
Age at survey, mean (SD), y	70.6 (7.3)	70.6 (7.2)	69.4 (6.8)	72.6 (7.7)
Aged ≥65 y	10 706 (72.6)	4693 (73.2)	3407 (67.9)	2606 (78.6)
Sex				
Female	8174 (55.4)	3245 (50.6)	2850 (56.8)	2079 (62.7)
Male	6575 (44.6)	3170 (49.4)	2170 (43.2)	1235 (37.3)
Educational attainment of college degree or higher	6931 (47.0)	2818 (43.9)	2758 (54.9)	1355 (40.9)
Missing	795 (5.4)	293 (4.6)	296 (5.9)	206 (6.2)
Born outside the US	9622 (65.2)	4312 (67.2)	4381 (87.3)	929 (28.0)
Missing	378 (2.6)	174 (2.7)	145 (2.9)	59 (1.8)
Household income per person, mean (SD), $	46 135 (28 114)	47 125 (29 342)	39 740 (24 809)	54 211 (28 147)
Missing	2117 (14.4)	860 (13.4)	691 (13.8)	566 (17.1)
Height, mean (SD), in	63.3 (3.2)	63.8 (3.2)	63.0 (3.1)	62.8 (3.3)
Missing	484 (3.3)	173 (2.7)	203 (4.0)	108 (3.3)
Size of household				
Living alone	2041 (13.8)	856 (13.3)	422 (8.4)	763 (23.0)
2 Individuals	4768 (32.3)	2185 (34.1)	1156 (23.0)	1427 (43.1)
≥3 Individuals	7429 (50.4)	3154 (49.2)	3254 (64.8)	1021 (30.8)
Missing	511 (3.5)	220 (3.4)	188 (3.7)	103 (3.1)
Married or living as if married	10 710 (72.6)	4944 (77.1)	3586 (71.4)	2180 (65.8)
Missing	162 (1.1)	91 (1.4)	43 (0.9)	28 (0.8)
Smoking status				
Never	9216 (62.5)	4320 (67.3)	3190 (63.5)	1706 (51.5)
Former	3514 (23.8)	1259 (19.6)	1062 (21.2)	1193 (36.0)
Current	648 (4.4)	257 (4.0)	228 (4.5)	163 (4.9)
Missing	1371 (9.3)	579 (9.0)	540 (10.8)	252 (7.6)
General health				
Excellent or very good	4093 (27.8)	1696 (26.4)	1257 (25.0)	1140 (34.4)
Good	6254 (42.4)	2723 (42.4)	2110 (42.0)	1421 (42.9)
Fair or poor	3478 (23.6)	1554 (24.2)	1297 (25.8)	627 (18.9)
Missing	924 (6.3)	442 (6.9)	356 (7.1)	126 (3.8)
Retired, yes	9675 (65.6)	4406 (68.7)	2908 (57.9)	2361 (71.2)
Self-reported stroke, yes	734 (5.0)	285 (4.4)	278 (5.5)	171 (5.2)
Self-reported hypertension, yes	6950 (47.1)	2786 (43.4)	2636 (52.5)	1528 (46.1)
Self-reported diabetes, yes	3104 (21.0)	1085 (16.9)	1373 (27.4)	646 (19.5)
End of follow-up event				
Administratively censored	7033 (47.7)	3303 (51.5)	2248 (44.8)	1482 (44.7)
Censored at age ≥90 y^a^	770 (5.2)	341 (5.3)	171 (3.4)	258 (7.8)
Death	2420 (16.4)	1066 (16.6)	766 (15.3)	588 (17.7)
Dementia	1895 (12.8)	769 (12.0)	566 (11.3)	560 (16.9)
End of membership	2631 (17.8)	936 (14.6)	1269 (25.3)	426 (12.9)
Follow-up time, mean (SD), y	9.9 (4.6)	10.5 (4.6)	9.3 (4.7)	9.8 (4.6)

SI conversion factor: To convert inches to centimeters, multiply by 2.54.

^a^ For deidentification purposes, censoring events after age 90 years were not differentiated between lapse in health plan membership and administrative censoring.

Overall, dementia incidence rates were higher among those with less than a college degree, with smaller differences after age standardization. Among individuals born outside the US in the sample, dementia incidence rates were 9.3 cases per 1000 person-years (PY) among those with a college degree and 14.7 cases per 1000 PY among those with less than a college degree. Similarly, among the individuals born in the US, dementia incidence rates were 10.8 cases per 1000 PY among those with a college degree and 19.2 cases per 1000 PY among those with less than a college degree. After age standardization, dementia incidence rates were 8.7 cases per 1000 PY among individuals born outside the US with a college degree, 10.7 cases per 1000 PY among individuals born outside the US without a college degree, 8.2 cases per 1000 PY among individuals born in the US with a college degree, and 10.4 cases per 1000 PY among individuals born in the US without a college degree. Ethnicity-specific dementia incidence rates are given in eTable 5 in Supplement 1. Absolute rates varied, but trends for education were similar across groups.

**Figure 1.**
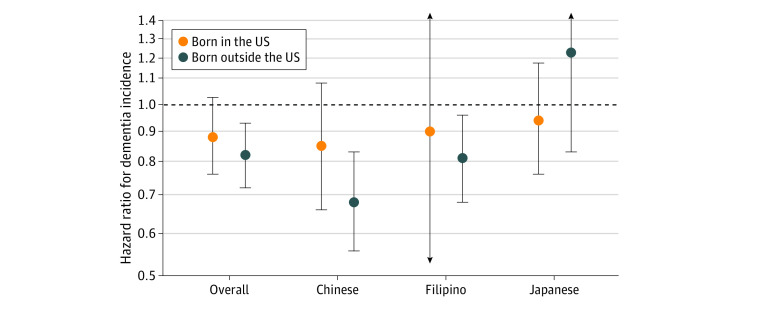
Hazard Ratios for the Association of Education Level (College Degree or Higher vs Less Than College Degree) With Dementia Incidence by Ethnicity and Nativity Cox proportional hazards models were adjusted for age (timescale) and sex. Error bars denote 95% CIs, and arrows indicate 95% CIs that extend beyond the scale of the figure (values are provided in Table 2).

In Cox proportional hazards models, college degree attainment was associated with lower dementia incidence among Asian American individuals born both in the US and born outside the US (Table 2 and Figure 1). Among individuals born in the US, those with a college degree had 12% lower dementia incidence (HR, 0.88; 95% CI, 0.75-1.03) compared with those without at least a college degree, although the confidence interval included the null. The HR for the individuals born outside the US was 0.82 (95% CI, 0.72-0.92; *P* = .46 for the college degree by nativity interaction). Although less precise, associations varied across Asian ethnicities, with larger nativity differences in the association between college degree and dementia among Chinese individuals (HR, 0.84 [95% CI, 0.65-1.08] for individuals born in the US; HR, 0.67; [95% CI, 0.55-0.82] for individuals born outside the US) than for Filipino or Japanese individuals. Of note, results for Filipino individuals born in the US and Japanese individuals born outside the US had wide 95% CIs that included both positive and negative associations.

**Table 2.** Hazard Ratios for the Association of Education With Dementia Incidence by Ethnicity and Nativity

Variable	Hazard ratio (95% CI)^a^			
	Overall (N = 14 749)	Chinese (n = 6415)	Filipino (n = 5020)	Japanese (n = 3314)
College education or higher in those born in the US	0.88 (0.75-1.03)	0.84 (0.65-1.08)	0.89 (0.43-1.87)	0.94 (0.75-1.17)
College education or higher in those born outside the US	0.82 (0.72-0.92)	0.67 (0.55-0.82)	0.80 (0.67-0.96)	1.23 (0.83-1.82)
*P* value for interaction	.46	.17	.78	.25

^a^ Cox proportional hazards models controlled for age (timescale) and sex.

**Figure 2.**
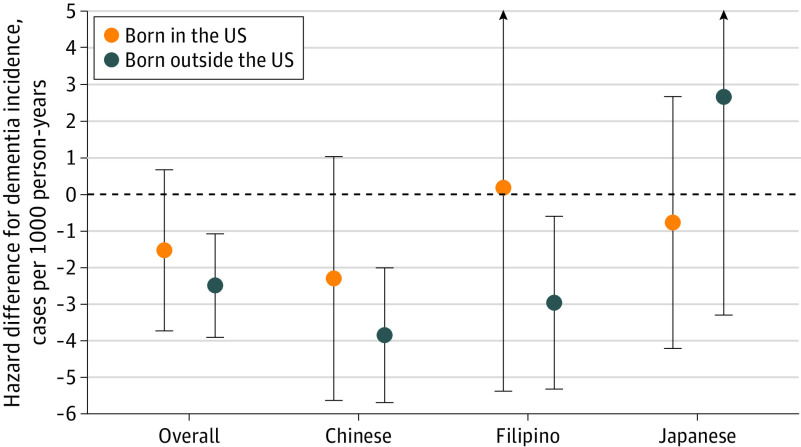
Hazard Differences for the Association of Education Level (College Degree or Higher vs Less Than College Degree) With Dementia Incidence by Ethnicity and Nativity Aalen additive hazards models were adjusted for age (timescale) and sex. Error bars denote 95% CIs, and arrows indicate 95% CIs that extend beyond the scale of the figure (values are provided in Table 3).

Associations on the additive scale showed similar trends (Table 3 and Figure 2). Overall, having a college degree was associated with fewer dementia cases per 1000 PY among Asian individuals born both inside and outside the US (HD, −1.53 cases per 1000 PY [95% CI, −3.73 to 0.67 cases per 1000 PY] among individuals born in the US; HD, −2.49 cases per 1000 PY [95% CI, −3.90 to −1.07 cases per 1000 PY] among individuals born outside the US; *P* = .47 for college degree by nativity interaction). The association between a college degree and lower dementia incidence was largest among Chinese individuals, and differences in associations by nativity were small across all ethnic groups. As with results on the HR scale, estimates in Filipino individuals born in the US and Japanese individuals born outside the US were particularly imprecise. Sensitivity analyses with (1) models additionally adjusted for height, (2) models using time on study and controlled for baseline age, and (3) models using a 3-level version of education yielded similar results (eTables 6-8 and eFigures 1 and 2 in Supplement 1).

**Table 3.** Hazard Differences for the Association of Education With Dementia Incidence by Ethnicity and Nativity

Variable	Hazard difference (95% CI)^a^			
	Overall (N = 14 749)	Chinese (n = 6415)	Filipino (n = 5020)	Japanese (n = 3314)
College education or higher in those born in the US	−1.53 (−3.73 to 0.67)	−2.30 (−5.63 to 1.02)	0.17 (−5.37 to 5.73)	−0.77 (−4.21 to 2.67)
College education or higher in those born outside the US	−2.49 (−3.90 to −1.07)	−3.85 (−5.69 to −2.01)	−2.96 (−5.32 to −0.59)	2.65 (−3.29 to 8.60)
*P* value for interaction	.47	.43	.31	.33

^a^ Aalen additive hazards models controlled for age (timescale) and sex.

## Discussion

The aim of this cohort study was to estimate the association of education and dementia incidence in a large cohort of Asian American individuals and to evaluate whether nativity modified this association. We found similar protective associations of having a college degree with dementia incidence by nativity on both multiplicative and additive scales and no clear differences across Asian ethnic groups, although estimates for Filipino individuals born in the US and Japanese individuals born outside the US were imprecise.

Despite the importance of education as a determinant of dementia and potential for heterogeneity in its impact on late-life cognitive function by race and ethnicity,^6,7^ to our knowledge, our study is the first to report the association between education and dementia incidence in multiple Asian American ethnic groups. We showed that college degree attainment vs less than a college degree was protective overall and for Chinese and Filipino individuals regardless of nativity. Among Japanese individuals born outside the US, dementia incidence was slightly higher in those with vs without a college degree, but the sample size of Japanese individuals born outside the US was small and the 95% CIs were wide, including both positive and negative associations. To our knowledge, there are no estimates of the association between education and dementia incidence in Chinese and Filipino American individuals. Prior work in the Kame cohort of Japanese American individuals in King County, Washington, showed that each additional year of education was associated with 6% lower incidence of dementia,^32^ but that study did not stratify by nativity. Although our estimates are slightly smaller than those in other literature, comparison of estimates across studies is complicated by heterogeneity in measurement and operationalization of educational attainment.^4^

Our work builds on other work showing differential returns on education for health among Asian American individuals and, to our knowledge, is the first to examine dementia outcomes. Reduced benefits of education for cardiovascular health have been shown among Asian American individuals born inside vs outside the US who were aged 25 years and older in California.^33^ Other work^11^ among Asian American individuals showed that higher education was associated with better self-rated health, but found differences in this association by country of origin and duration in the US (eg, stronger educational gradients in self-rated health among Filipino individuals born inside vs outside the US, but no educational gradient for Japanese individuals born in the US). In contrast, we did not observe substantial variability across nativity. We examined both multiplicative and additive scales to ensure that our conclusions were not scale dependent^34^ and conducted a sensitivity analysis with a 3-level version of educational attainment. The lack of differences in educational benefits by nativity could reflect true robustness of the education-dementia association, or could reflect bias resulting from differences in education-related factors (eg, whether education occurred in the US or internationally, which likely varies by ethnicity, and measures of quality of education were not collected in the surveys) or different sources of confounding (eg, selective migration by those with social advantage). Understanding the mechanisms through which education is protective against dementia (eg, through mediation analyses), was beyond the scope of this article, but is an important next step for this work. Measures of potential mediators in this data set have limitations; for example, although income across the life course may mediate the impact of education on dementia risk through conferring financial resources, income measured at a single time point in late life may have different meanings depending on retirement status and sources of income and may not clearly capture financial advantage.^35^

### Limitations

Limitations of the study include lack of data on immigration-related details (eg, age of or reason for migration). Historical patterns and reasons for immigration vary across Asian ethnic groups, and we could not assess the potential association of these factors with dementia incidence in the individuals born outside the US.^25^ Most Japanese American individuals were born in the US and are the descendants of immigrants who arrived in the US before the passage of the 1924 National Origins Act, which largely prohibited immigration from Asian countries, and most Chinese and Filipino American individuals arrived in the US after 1965 because of major changes to US immigration policies with the passage of the Immigration and Nationality Act.^36^ We also did not have information on languages spoken by participants or specific early-life confounders of the education-dementia association. However, in sensitivity analyses, we were able to adjust for height, which may be a proxy for a variety of potential early-life confounders (eg, socioeconomic status).^26,27,28^

In addition, our time-to-dementia outcome was derived from dementia diagnoses in the electronic health record, rather than from data collected specifically for research purposes. Dementia diagnosis may be subject to misclassification, and if missed diagnoses were differential by educational attainment (eg, more missed cases in low education groups, as has been shown among Medicare beneficiaries^37^), our estimates of the protective association with educational attainment would be conservative (biased toward the null). Similarly, if dementia diagnoses were more likely to be missed among individuals born outside the US, particularly individuals born outside the US without a college degree, we would expect our estimates of the protective association of educational attainment would be particularly biased toward the null in the individuals born outside the US, which would strengthen our finding that associations between education and dementia do not appear to be attenuated in Asian American individuals born outside the US vs those born in the US. In addition, KPNC members all have health insurance (although we did not have measures of insurance type, such as Medicare vs employer-based, that could provide further insight on social advantage), and prior work^25^ in this survey cohort has shown that participants were healthier and more likely to be English-speaking (surveys were administered in English, Spanish, and Chinese) than the California general population of older adults, which may limit the generalizability of our results to this population.

## Conclusions

Asian American individuals remain a critically understudied population in both dementia research and health research more broadly. This study, the first to our knowledge to examine the association of educational attainment with dementia incidence across multiple Asian ethnic groups, showed that education is an important modifiable determinant of dementia in Asian American individuals, with similar associations in individuals born outside the US vs individuals born in the US. Future work should focus on understanding the reasons for the lower dementia incidence rates among Asian American individuals vs other racial and ethnic groups, including both distribution of risk and protective factors and potential differential impact, and continue to explore the pathways by which education impacts dementia risk, including mediation by social factors such as income and impacts on cognitive reserve and resilience.

## Notes

Supplement 1. **eTable 1.** *International Classification of Disease, Ninth Edition, Clinical Modification (ICD-9-CM) *and *International Classification of Disease, Tenth Edition, Clinical Modification (ICD-10-CM) *Codes Used to Identify Dementia (Alzheimer’s Disease, Vascular Dementia, and Nonspecific Dementia) **eAppendix 1.** Additional Details On Height Variable **eAppendix 2.** Imputation Approach **eTable 2.** Percent Missing Values by Ethnicity for Variables in Table 1 **eTable 3.** Baseline Sample Characteristics Stratified by Ethnicity and Educational Attainment (Postimputation) **eTable 4.** Baseline Sample Characteristics Stratified by Ethnicity and Nativity (Postimputation) **eTable 5.** Ethnicity-Specific Crude and Age-Standardized Dementia Incidence Rates **eTable 6.** Sensitivity Analysis Adjusting for Age (Timescale), Sex/Gender, and Height **eTable 7.** Sensitivity Analysis Using Time on Study and Adjusting for Baseline Age and Sex/Gender **eTable 8.** Sensitivity Analysis With Three-Level Education Variable, Adjusted for Age (Timescale) and Sex/Gender **eFigure 1.** Hazard Ratios Relating Three-Level Education Variable and Dementia Incidence by Ethnicity and Nativity Adjusted for Age (Timescale) and Sex/Gender **eFigure 2.** Hazard Differences Relating Three-Level Education Variable and Dementia Incidence by Ethnicity and Nativity, Adjusted for Age (Timescale) and Sex/Gender
eReference

Supplement 2.
Data Sharing Statement
