# Supplementary material for: Association of Education With Dementia Incidence Stratified by Ethnicity and Nativity in a Cohort of Older Asian American Individuals
Source: JAMA Netw Open. 2023 Mar 6;6(3):e231661. doi: 10.1001/jamanetworkopen.2023.1661 (PMC9989900; doi:10.1001/jamanetworkopen.2023.1661)
Supplement: Supplement 1. — eTable 1. International Classification of Disease, Ninth Edition, Clinical Modification (ICD-9-CM) and International Classification of Disease, Tenth Edition, Clinical Modification (ICD-10-CM) Codes Used to Identify Dementia (Alzheimer’s Disease, Vascular Dementia, and Nonspecific Dementia) eAppendix 1. Additional Details On Height Variable eAppendix 2. Imputation Approach eTable 2. Percent Missing Values by Ethnicity for Variables in Table 1 eTable 3. Baseline Sample Characteristics Stratified by Ethnicity and Educational Attainment (Postimputation) eTable 4. Baseline Sample Characteristics Stratified by Ethnicity and Nativity (Postimputation) eTable 5. Ethnicity-Specific Crude and Age-Standardized Dementia Incidence Rates eTable 6. Sensitivity Analysis Adjusting for Age (Timescale), Sex/Gender, and Height eTable 7. Sensitivity Analysis Using Time on Study and Adjusting for Baseline Age and Sex/Gender eTable 8. Sensitivity Analysis With Three-Level Education Variable, Adjusted for Age (Timescale) and Sex/Gender eFigure 1. Hazard Ratios Relating Three-Level Education Variable and Dementia Incidence by Ethnicity and Nativity Adjusted for Age (Timescale) and Sex/Gender eFigure 2. Hazard Differences Relating Three-Level Education Variable and Dementia Incidence by Ethnicity and Nativity, Adjusted for Age (Timescale) and Sex/Gender eReference [file jamanetwopen-e231661-s001.pdf]

Supplemental Online Content

Hayes-Larson E, Ikesu R, Fong J, et al. Association of education with dementia incidence stratified by ethnicity and nativity in a cohort of older Asian American individuals. *JAMA Netw Open*. 2023;6(3):e231661. doi:10.1001/jamanetworkopen.2023.1661

eTable 1. *International Classification of Disease, Ninth Edition, Clinical Modification (ICD-9-CM)* and *International Classification of Disease, Tenth Edition, Clinical Modification (ICD-10-CM)* Codes Used to Identify Dementia (Alzheimer's Disease, Vascular Dementia, and Nonspecific Dementia)

eAppendix 1. Additional Details On Height Variable

eAppendix 2. Imputation Approach

eTable 2. Percent Missing Values by Ethnicity for Variables in Table 1

eTable 3. Baseline Sample Characteristics Stratified by Ethnicity and Educational Attainment (Postimputation)

eTable 4. Baseline Sample Characteristics Stratified by Ethnicity and Nativity (Postimputation)

eTable 5. Ethnicity-Specific Crude and Age-Standardized Dementia Incidence Rates

eTable 6. Sensitivity Analysis Adjusting for Age (Timescale), Sex/Gender, and Height

eTable 7. Sensitivity Analysis Using Time on Study and Adjusting for Baseline Age and Sex/Gender

eTable 8. Sensitivity Analysis With Three-Level Education Variable, Adjusted for Age (Timescale) and Sex/Gender

eFigure 1. Hazard Ratios Relating Three-Level Education Variable and Dementia Incidence by Ethnicity and Nativity Adjusted for Age (Timescale) and Sex/Gender

eFigure 2. Hazard Differences Relating Three-Level Education Variable and Dementia Incidence by Ethnicity and Nativity, Adjusted for Age (Timescale) and Sex/Gende2

eReference

This supplemental material has been provided by the authors to give readers additional information about their work.

© 2023 Hayes-Larson E et al. *JAMA Network Open.*

eTable 1. *International Classification of Disease, Ninth Edition, Clinical Modification (ICD-9-CM)* and *International Classification of Disease, Tenth Edition, Clinical Modification (ICD-10-CM)* Codes Used to Identify Dementia (Alzheimer's Disease, Vascular Dementia, and Nonspecific Dementia)

	**ICD Code**	**Description**
ICD-9-CM	331.0	Alzheimer's disease
	290.40	Vascular dementia, uncomplicated
	290.41	Vascular dementia, with delirium
	290.42	Vascular dementia, with delusions
	290.43	Vascular dementia, with depressed mood
	290.0	Senile dementia, uncomplicated
	290.10	Presenile dementia, uncomplicated
	290.11	Presenile dementia with delirium
	290.12	Presenile dementia with delusional features
	290.13	Presenile dementia with depressive features
	290.3	Senile dementia with delirium
	294.20	Dementia, unspecified, without behavioral disturbance
	294.21	Dementia, unspecified, with behavioral disturbance
	294.8	Other persistent mental disorders due to conditions classified elsewhere
ICD-10-CM	G30.0	Alzheimer's Disease with early onset
	G30.1	Alzheimer's disease with late onset
	G30.8	Other Alzheimer's disease
	G30.9	Alzheimer's disease, unspecified
	F01.50	Vascular dementia without behavioral disturbance
	F01.51	Vascular dementia with behavioral disturbance
	F03.90	Unspecified Dementia without behavioral disturbance
	F03.91	Unspecified Dementia with behavioral disturbance

© 2023 Hayes-Larson E et al. *JAMA Network Open.*

eAppendix 1. Additional Details On Height Variable

Height was obtained from electronic health records as follows. The data contained median annual height measures from 1/1/2005 (when height started being routinely recorded in the Kaiser Permanente Northern California electronic health records) until participant death or censoring, whichever occurred first. Participants with only one annual median height (i.e., median height for a single year) were assigned that value as their “main adult height.” For participants with multiple annual median height measures, we estimated their “main adult height” as (A) the annual median height mode, if a unique mode existed, (B) the first mode if multiple modes existed, or (C) the first recorded median height if all values were different.

eAppendix 2. Imputation Approach

eTable 2 shows the proportion of respondents missing data on the analytic and descriptive variables in this paper. Missing data were handled with multiple imputation using chained equations and predictive means matching for continuous variables, logistic regression for binary variables, polytomous logistic regression for categorical variables, and ordinal regression for ordered categorical variables.[1] Imputation models included all variables with missingness shown in eTable 2. Auxiliary variables used to aid imputation of the variables in eTable 2 included 7 completely-observed variables (baseline age, sex/gender, ethnicity, language of survey administration [Chinese or English], and self-reported history of stroke, diabetes, and hypertension at baseline), and 3 additional variables with some missingness: father’s nativity (2.1% missing), mother’s nativity (1.7% missing), and household income (13.1% missing).

eTable 2. Percent Missing Values by Ethnicity for Variables in Table 1

**Variable**	**Chinese N = 6415**	**Filipino N = 5020**	**Japanese N = 3314**
Analytic variables			
US-born	2.7%	2.8%	1.7%
Educational attainment	4.5%	5.8%	6.2%
Descriptive variables			
Household income per person	13.4%	13.8%	17.1%
Height	2.7%	4.0%	3.3%
Size of household	3.4%	3.7%	3.1%
Marital status	1.4%	0.8%	0.8%
Smoking status	9.0%	10.7%	7.6%
General health	6.8%	7.0%	3.8%

© 2023 Hayes-Larson E et al. *JAMA Network Open.*

eTable 3. Baseline Sample Characteristics Stratified by Ethnicity and Educational Attainment (Postimputation)[a]

	Overall N = 14,749		Chinese N = 6415		Filipino N = 5020		Japanese N= 3314	
	Less than college degree N = 7468 (50.6)	College degree or higher N = 7281 (49.4)	Less than college degree N = 3483 (54.3)	College degree or higher N = 2932 (45.7)	Less than college degree N = 2112 (42.1)	College degree or higher N = 2908 (57.9)	Less than college degree N = 1873 (56.6)	College degree or higher N = 1441 (43.4)
**Survey age, years (mean [SD])**	71.8 (7.5)	69.4 (6.9)	71.4 (7.3)	69.6 (6.9)	70.4 (7.0)	68.7 (6.5)	74.3 (7.5)	70.4 (7.3)
**65 years or older (%)**	5790 (77.5)	4916 (67.5)	2668 (76.6)	2025 (69.0)	1526 (72.2)	1881 (64.7)	1595 (85.2)	1011 (70.1)
**Sex/gender (%)**								
Female	4371 (58.5)	3803 (52.2)	1905 (54.7)	1340 (45.7)	1168 (55.3)	1682 (57.8)	1298 (69.3)	781 (54.2)
Male	3097 (41.5)	3478 (47.8)	1578 (45.3)	1592 (54.3)	944 (44.7)	1226 (42.2)	575 (30.7)	660 (45.8)
**Household income per person (USD, mean [SD])**	37410 (24805)	53728 (28869)	37059 (25257)	57731 (29699)	31697 (21756)	44207 (25366)	44506 (25416)	64789 (27926)
**Foreign born (%)**	4908 (65.7)	4990 (68.5)	2499 (71.7)	1944 (66.3)	1764 (83.5)	2745 (94.4)	645 (34.5)	301 (20.9)
**Height (inch, mean [SD])**	63.0 (3.2)	63.7 (3.3)	63.4 (3.1)	64.4 (3.2)	63.0 (3.1)	63.1 (3.1)	62.3 (3.1)	63.5 (3.4)
**Size of household (%)**								
Living alone	1180 (15.8)	945 (13.0)	501 (14.4)	392 (13.4)	193 (9.2)	244 (8.4)	486 (26.0)	309 (21.4)
Two	2333 (31.2)	2616 (35.9)	1062 (30.5)	1208 (41.2)	492 (23.3)	717 (24.7)	779 (41.6)	690 (47.9)
Three or more	3954 (53.0)	3721 (51.1)	1920 (55.1)	1332 (45.4)	1427 (67.5)	1946 (66.9)	608 (32.5)	443 (30.7)
**Married/living as married (%)**	5251 (70.3)	5566 (76.4)	2647 (76.0)	2359 (80.4)	1442 (68.3)	2173 (74.7)	1162 (62.0)	1034 (71.7)
**Smoking status (%)**								
Never	4920 (65.9)	5242 (72.0)	2525 (72.5)	2230 (76.0)	1365 (64.6)	2201 (75.7)	1030 (55.0)	811 (56.3)
Former	2068 (27.7)	1797 (24.7)	754 (21.7)	622 (21.2)	596 (28.2)	597 (20.5)	718 (38.4)	578 (40.1)
Current	480 (6.4)	242 (3.3)	204 (5.8)	81 (2.8)	152 (7.2)	109 (3.8)	124 (6.6)	52 (3.6)
**General health (%)**								
Excellent/very good	1856 (24.9)	2515 (34.5)	792 (22.7)	1034 (35.2)	522 (24.7)	834 (28.7)	542 (29.0)	648 (44.9)
Good	3384 (45.3)	3285 (45.1)	1581 (45.4)	1335 (45.5)	919 (43.5)	1360 (46.8)	884 (47.2)	591 (41.0)
Fair/poor	2228 (29.8)	1480 (20.3)	1110 (31.9)	564 (19.2)	671 (31.8)	714 (24.5)	447 (23.9)	202 (14.0)
**Retired, yes (%)**	5038 (67.5)	4637 (63.7)	2421 (69.5)	1985 (67.7)	1285 (60.8)	1623 (55.8)	1333 (71.2)	1028 (71.4)
**Self-reported stroke (%)**	399 (5.3)	335 (4.6)	162 (4.6)	124 (4.2)	129 (6.1)	149 (5.1)	108 (5.8)	63 (4.4)
**Self-reported hypertension (%)**	3512 (47.0)	3439 (47.2)	1534 (44.1)	1252 (42.7)	1090 (51.6)	1546 (53.2)	888 (47.4)	641 (44.4)
**Self-reported diabetes (%)**	1533 (20.5)	1571 (21.6)	610 (17.5)	475 (16.2)	563 (26.6)	810 (27.9)	360 (19.2)	286 (19.8)
**End of follow up event (%)**								
Administratively censored	3166 (42.4)	3867 (53.1)	1641 (47.1)	1662 (56.7)	829 (39.2)	1419 (48.8)	696 (37.2)	786 (54.5)
Censored 90+[b]	477 (6.4)	293 (4.0)	202 (5.8)	139 (4.7)	88 (4.1)	84 (2.9)	187 (10.0)	71 (4.9)
Death	1374 (18.4)	1046 (14.4)	641 (18.4)	425 (14.5)	346 (16.4)	421 (14.5)	387 (20.7)	201 (14.0)
Dementia	1172 (15.7)	723 (9.9)	507 (14.6)	262 (8.9)	292 (13.8)	274 (9.4)	372 (19.9)	188 (13.0)
End of membership	1280 (17.1)	1351 (18.6)	491 (14.1)	445 (15.2)	558 (26.4)	711 (24.4)	231 (12.3)	195 (13.6)
**Follow-up time, years (mean [SD])**	9.6 (4.7)	10.2 (4.6)	10.2 (4.7)	10.8 (4.5)	9.0 (4.7)	9.5 (4.6)	9.3 (4.6)	10.5 (4.4)

[a]Decimals for N's reflect that the table averages the number of people across imputed datasets.

[b]For de-identification purposes, censoring events after age 90 were not differentiated between lapse in health plan membership and administrative censoring.

Abbreviations: SD = standard deviation, USD = US dollars.

© 2023 Hayes-Larson E et al. *JAMA Network Open.*

eTable 4. Baseline Sample Characteristics Stratified by Ethnicity and Nativity (Postimputation)[a]

	Overall		Chinese		Filipino		Japanese	
	**US-born N = 4851**	**Foreign-born N = 9898**	**US-born N = 1973**	**Foreign-born N = 4442**	**US-born N = 511**	**Foreign-born N = 4509**	**US-born N = 2368**	**Foreign-born N = 946**
**Survey age, years (mean [SD])**	72.2 (7.9)	69.9 (6.8)	72.1 (7.8)	69.9 (6.8)	69.1 (6.7)	69.4 (6.8)	72.9 (8.1)	71.9 (6.6)
**65 years or older (%)**	3690 (76.1)	7016 (70.9)	1515 (76.8)	3178 (71.5)	333 (65.2)	3074 (68.2)	1842 (77.8)	765 (80.8)
**Sex/gender (%)**								
Female	2664 (54.9)	5510 (55.7)	1072 (54.3)	2173 (48.9)	292 (57.1)	2558 (56.7)	1300 (54.9)	779 (82.3)
Male	2187 (45.1)	4388 (44.3)	901 (45.7)	2269 (51.1)	219 (42.9)	1951 (43.3)	1068 (45.1)	167 (17.7)
**Household income per person (USD, mean [SD])**	56930 (28353)	39846 (26201)	58944 (28375)	40987 (27907)	49156 (26216)	37785 (24251)	56930 (28490)	44311 (25994)
**Educational attainment, ≥ college degree, (%)**	2291 (47.2)	4990 (50.4)	989 (50.1)	1944 (43.8)	163 (31.8)	2745 (60.9)	1140 (48.2)	301 (31.8)
**Height (inch, mean [SD])**	63.6 (3.4)	63.2 (3.1)	64.0 (3.4)	63.8 (3.1)	63.9 (3.5)	63.0 (3.1)	63.3 (3.4)	61.7 (2.7)
**Size of household (%)**								
Living alone	1051 (21.7)	1074 (10.9)	414 (21.0)	479 (10.8)	90 (17.6)	347 (7.7)	547 (23.1)	248 (26.2)
Two	2217 (45.7)	2732 (27.6)	914 (46.3)	1357 (30.5)	206 (40.3)	1004 (22.3)	1097 (46.3)	371 (39.2)
Three or more	1584 (32.6)	6092 (61.5)	645 (32.7)	2607 (58.7)	215 (42.1)	3158 (70.0)	723 (30.5)	327 (34.6)
**Married/living as married (%)**	3326 (68.6)	7490 (75.7)	1376 (69.7)	3630 (81.7)	344 (67.2)	3272 (72.6)	1607 (67.9)	588 (62.2)
**Smoking status (%)**								
Never	2761 (56.9)	7402 (74.8)	1288 (65.3)	3467 (78.0)	244 (47.7)	3322 (73.7)	1229 (51.9)	613 (64.7)
Former	1839 (37.9)	2026 (20.5)	604 (30.6)	772 (17.4)	223 (43.6)	970 (21.5)	1012 (42.8)	284 (30.0)
Current	251 (5.2)	470 (4.7)	81 (4.1)	203 (4.6)	44 (8.7)	217 (4.8)	126 (5.3)	50 (5.3)
**General health (%)**								
Excellent/very good	1784 (36.8)	2587 (26.1)	718 (36.4)	1108 (24.9)	174 (34.1)	1182 (26.2)	892 (37.7)	298 (31.4)
Good	2211 (45.6)	4458 (45.0)	927 (47.0)	1989 (44.8)	227 (44.5)	2051 (45.5)	1057 (44.6)	418 (44.1)
Fair/poor	856 (17.6)	2852 (28.8)	328 (16.6)	1346 (30.3)	109 (21.4)	1276 (28.3)	418 (17.7)	231 (24.4)
**Retired, yes (%)**	3703 (76.3)	5972 (60.3)	1534 (77.8)	2872 (64.6)	349 (68.2)	2559 (56.8)	1820 (76.9)	541 (57.1)
**Self-reported stroke (%)**	240 (5.0)	494 (5.0)	91 (4.6)	194 (4.4)	26 (5.0)	253 (5.6)	123 (5.2)	48 (5.0)
**Self-reported hypertension (%)**	2420 (49.9)	4530 (45.8)	982 (49.8)	1804 (40.6)	273 (53.5)	2363 (52.4)	1165 (49.2)	363 (38.4)
**Self-reported diabetes (%)**	972 (20.0)	2133 (21.5)	351 (17.8)	734 (16.5)	129 (25.2)	1244 (27.6)	492 (20.8)	154 (16.3)
**End of follow up event (%)**								
Administratively censored	2268 (46.7)	4765 (48.1)	960 (48.6)	2343 (52.8)	257 (50.2)	1992 (44.2)	1052 (44.4)	430 (45.5)
Censored 90+[b]	376 (7.8)	394 (4.0)	164 (8.3)	177 (4.0)	22 (4.3)	149 (3.3)	190 (8.0)	68 (7.1)
Death	940 (19.4)	1480 (14.9)	395 (20.0)	671 (15.1)	89 (17.4)	677 (15.0)	457 (19.3)	131 (13.8)
Dementia	732 (15.1)	1163 (11.7)	286 (14.5)	483 (10.9)	46 (9.1)	520 (11.5)	400 (16.9)	160 (16.9)
End of membership	535 (11.0)	2096 (21.2)	169 (8.5)	767 (17.3)	97 (19.1)	1172 (26.0)	269 (11.4)	157 (16.6)
**Follow-up time, years (mean [SD])**	10.1 (4.6)	9.8 (4.7)	10.5 (4.5)	10.4 (4.6)	10.0 (4.7)	9.2 (4.6)	9.8 (4.6)	9.9 (4.4)

[a]Decimals for N's reflect that the table averages the number of people across imputed datasets.

[b]For de-identification purposes, censoring events after age 90 were not differentiated between lapse in health plan membership and administrative censoring.

Abbreviations: SD = standard deviation, USD = US dollars.

© 2023 Hayes-Larson E et al. *JAMA Network Open.*

eTable 5. Ethnicity-Specific Crude and Age-Standardized Dementia Incidence Rates[a]

Ethnicity & College status	Nativity Status							
	US-Born				Foreign-born			
	Events	PYs	Crude IR (95% CI)	Age-standardized IR (95% CI)	Events	PYs	Crude IR (95% CI)	Age-standardized IR (95% CI)
Chinese ≥College	113	10971.6	10.3 (8.4, 12.2)	7.51 (6.12, 8.9)	154	20865.5	7.38 (6.21, 8.55)	6.62 (5.52, 7.72)
Chinese <College	175	9791.3	17.87 (15.22, 20.52)	10.45 (8.12, 12.78)	327	25523.6	12.81 (11.42, 14.20)	9.80 (8.70, 10.90)
Filipino ≥College	10	1675.9	5.97 (2.27, 9.67)	7.36 (2.22, 12.5)	264	25963.6	10.17 (8.94, 11.40)	10.13 (8.84, 11.42)
Filipino <College	35	3389.2	10.33 (6.91, 13.75)	8.54 (5.7, 11.38)	257	15605.4	16.47 (14.46, 18.48)	12.22 (10.71 ,13.73)
Japanese ≥College	143	12104.4	11.81 (9.87, 13.75)	8.83 (7.37, 10.29)	43	2940.9	14.62 (10.25, 18.99)	11.77 (8.17, 15.37)
Japanese <College	259	11257.1	23.01 (20.21, 25.81)	10.87 (9.14, 12.6)	115	6302.6	18.25 (14.92, 21.58)	10.57 (8.51, 12.63)
Overall ≥College	266	24752.0	10.75 (9.46, 12.04)	8.15 (7.16, 9.14)	461	49769.9	9.26 (8.41, 10.11)	8.72 (7.88, 9.56)
Overall <College	469	24437.6	19.19 (17.45, 20.93)	10.36 (9.16, 11.56)	699	47431.6	14.74 (13.65, 15.83)	10.66 (9.85, 11.47)

Abbreviations: PYs = person-years, IR = incidence rate, CI = confidence interval.

[a]Using 2000 US Census population as the standard population.

© 2023 Hayes-Larson E et al. *JAMA Network Open.*

eTable 6. Sensitivity Analysis Adjusting for Age (Timescale), Sex/Gender, and Height

	Overall N = 14,749		Chinese N = 6415		Filipino N = 5020		Japanese N = 3314	
Cox proportional hazards model results	***HR***	***95% CI***	***HR***	***95% CI***	***HR***	***95% CI***	***HR***	***95% CI***
College degree or higher in US born	0.89	(0.76, 1.04)	0.85	(0.66, 1.08)	0.89	(0.43, 1.85)	0.96	(0.77, 1.19)
College degree or higher in foreign born	0.82	(0.73, 0.93)	0.68	(0.56, 0.82)	0.80	(0.67, 0.96)	1.23	(0.83, 1.83)
P-value for interaction	0.46		0.17		0.78		0.27	
Aalen additive hazard model results	***HD***	***95% CI***	***HD***	***95% CI***	***HD***	***95% CI***	***HD***	***95% CI***
College degree or higher in US born	-1.51	(-3.71, 0.69)	-2.30	(-5.63, 1.02)	0.06	(-5.49, 5.61)	-0.58	(-4.03, 2.87)
College degree or higher in foreign born	-2.47	(-3.88, -1.05)	-3.86	(-5.70, -2.02)	-3.04	(-5.40, -0.68)	2.74	(-3.21, 8.69)
P-value for interaction	0.47		0.43		0.32		0.35	

eTable 7. Sensitivity Analysis Using Time on Study and Adjusting for Baseline Age and Sex/Gender

	Overall N = 14,749		Chinese N = 6415		Filipino N = 5020		Japanese N = 3314	
Cox proportional hazards model results	***HR***	***95% CI***	***HR***	***95% CI***	***HR***	***95% CI***	***HR***	***95% CI***
College degree or higher in US born	0.89	(0.76, 1.05)	0.84	(0.66, 1.09)	0.86	(0.41, 1.81)	0.99	(0.79, 1.23)
College degree or higher in foreign born	0.81	(0.72, 0.92)	0.68	(0.55, 0.82)	0.79	(0.66, 0.95)	1.23	(0.84, 1.82)
P-value for interaction	0.37		0.18		0.84		0.33	
Aalen additive hazard model results	***HD***	***95% CI***	***HD***	***95% CI***	***HD***	***95% CI***	***HD***	***95% CI***
College degree or higher in US born	-2.24	(-4.42, -0.05)	-2.06	(-5.39, 1.27)	-0.76	(-6.36, 4.83)	-1.38	(-4.86, 2.10)
College degree or higher in foreign born	-2.09	(-3.51, -0.67)	-3.58	(-5.43, -1.72)	-3.39	(-5.77, -1.00)	4.38	(-1.56, 10.33)
P-value for interaction	0.92		0.44		0.40		0.10	

© 2023 Hayes-Larson E et al. *JAMA Network Open.*

eTable 8. Sensitivity Analysis With Three-Level Education Variable, Adjusted for Age (Timescale) and Sex/Gender

	Overall N = 14,749		Chinese N = 6415		Filipino N = 5020		Japanese N = 3314	
Cox proportional hazards model results	***HR***	***95% CI***	***HR***	***95% CI***	***HR***	***95% CI***	***HR***	***95% CI***
US born								
College degree or higher	0.90	(0.77, 1.06)	0.87	(0.68, 1.13)	0.94	(0.43, 2.04)	0.96	(0.77, 1.20)
High school degree, GED, technical/trade school, or some college	Ref	--	Ref	--	Ref	--	Ref	--
Less than high school degree	1.21	(0.92, 1.59)	1.36	(0.84, 2.18)	1.16	(0.57, 2.38)	1.16	(0.76, 1.78)
Foreign born								
College degree or higher	0.83	(0.73, 0.96)	0.72	(0.58, 0.90)	0.78	(0.64, 0.95)	1.28	(0.85, 1.93)
High school degree, GED, technical/trade school, or some college	Ref	--	Ref	--	Ref	--	Ref	--
Less than high school degree	1.04	(0.89, 1.22)	1.14	(0.91, 1.43)	0.90	(0.70, 1.20)	1.15	(0.74, 1.79)
P-value for interaction								
(US born)*(College education or higher)	0.45		0.27		0.65		0.23	
(US born)*(Less than high school degree)	0.35		0.53		0.54		0.96	
Aalen additive hazard model results	***HD***	***95% CI***	***HD***	***95% CI***	***HD***	***95% CI***	***HD***	***95% CI***
US born								
College degree or higher	-1.15	(-3.37, 1.08)	-1.71	(-5.04, 1.63)	0.07	(-5.41, 5.55)	-0.49	(-3.95, 2.96)
High school degree, GED, technical/trade school, or some college	Ref	--	Ref	--	Ref	--	Ref	--
Less than high school degree	5.74	(-3.45, 14.92)	9.22	(-5.69, 24.14)	-0.93	(-15.93, 14.06)	5.96	(-11.46, 23.39)
Foreign born								
College degree or higher	-2.13	(-3.68, -0.58)	-2.94	(-5.02, -0.85)	-3.09	(-5.66, -0.52)	3.28	(-2.79, 9.36)
High school degree, GED, technical/trade school, or some college	Ref	--	Ref	--	Ref	--	Ref	--
Less than high school degree	1.14	(-1.41, 3.68)	2.34	(-0.71, 5.40)	-0.53	(-5.81, 4.74)	3.06	(-6.46, 12.58)
P-value for interaction								
(US born)*(College degree or higher)	0.48		0.54		0.31		0.29	
(US born)*(Less than high school degree)	0.34		0.38		0.96		0.78	

© 2023 Hayes-Larson E et al. *JAMA Network Open.*

eFigure 1. Hazard Ratios Relating Three-Level Education Variable and Dementia Incidence by Ethnicity and Nativity Adjusted for Age (Timescale) and Sex/Gender. Arrows indicate confidence intervals that extend beyond the scale of the figure (values provided in eTable 8).

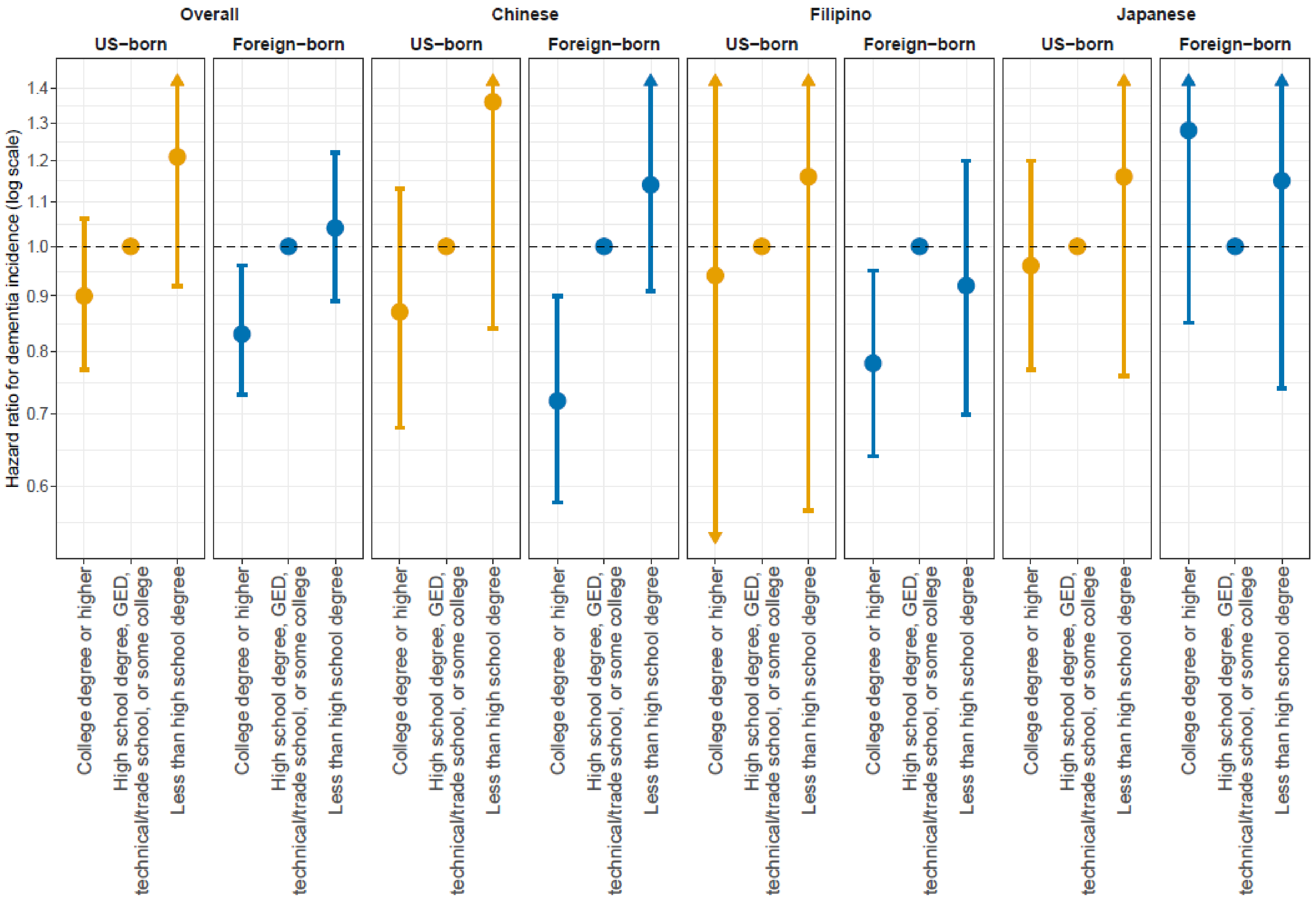

© 2023 Hayes-Larson E et al. *JAMA Network Open.*

eFigure 2. Hazard Differences Relating Three-Level Education Variable and Dementia Incidence by Ethnicity and Nativity, Adjusted for Age (Timescale) and Sex/Gender. Arrows indicate confidence intervals that extend beyond the scale of the figure (values provided in eTable 8).

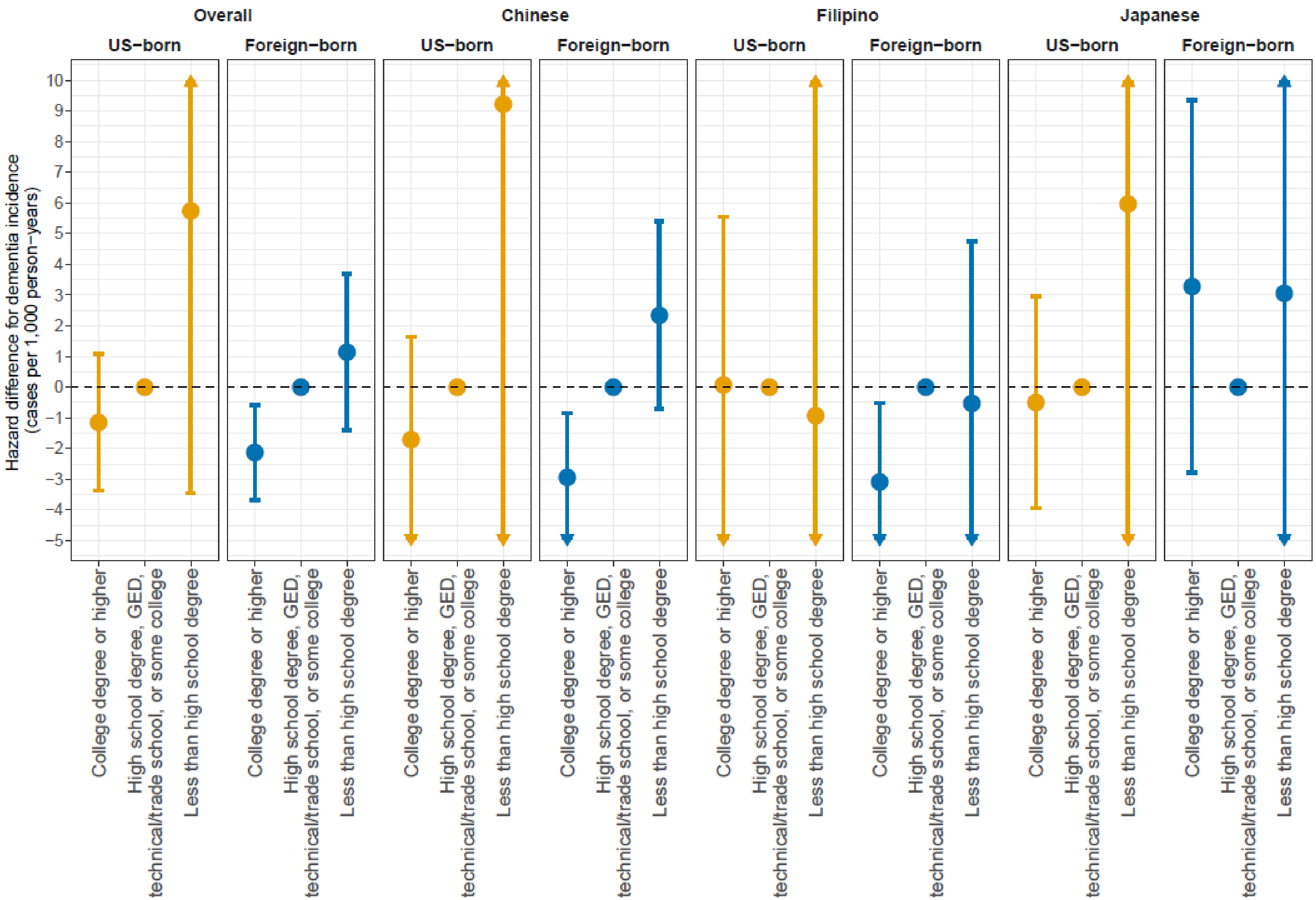

© 2023 Hayes-Larson E et al. *JAMA Network Open.*

eReference

1. Buuren S van. Flexible Imputation of Missing Data. Second Edition. Boca Raton, FL: CRC Press; 2018.

© 2023 Hayes-Larson E et al. *JAMA Network Open.*
