# Supplementary material for: Association of Education With Dementia Incidence Stratified by Ethnicity and Nativity in a Cohort of Older Asian American Individuals
Source: JAMA Netw Open. 2023 Mar 6;6(3):e231661. doi: 10.1001/jamanetworkopen.2023.1661 (PMC9989900; doi:10.1001/jamanetworkopen.2023.1661)
Supplement: Supplement 2. — Data Sharing Statement [file jamanetwopen-e231661-s002.pdf]

Data Sharing Statement

Hayes-Larson. Association of Education With Dementia Incidence Stratified by Ethnicity and Nativity in a Cohort of Older Asian American Individuals. *JAMA Netw Open*. Published March 06, 2023. doi:10.1001/jamanetworkopen.2023.1661

Data
Data available: No

Additional Information
Explanation for why data not available: The data underlying this article were provided by the Kaiser Permanente Research Bank. Information on the application process for researchers interested in using Kaiser Permanente Research Bank data can be found here: https://researchbank.kaiserpermanente.org/our-research/for-researchers/.
